# Pyrethroid resistance in *Anopheles gambiae *leads to increased susceptibility to the entomopathogenic fungi *Metarhizium anisopliae *and *Beauveria bassiana*

**DOI:** 10.1186/1475-2875-9-168

**Published:** 2010-06-16

**Authors:** Annabel FV Howard, Constantianus JM Koenraadt, Marit Farenhorst, Bart GJ Knols, Willem Takken

**Affiliations:** 1 Laboratory of Entomology, Wageningen University and Research Centre, P.O. Box 8031, 6700 EH Wageningen, The Netherlands; 2 Div. Infectious Diseases, Tropical Medicine & AIDS, Academic Medical Center, F4-217 Meibergdreef 9, 1105 AZ Amsterdam, The Netherlands

## Abstract

**Background:**

Entomopathogenic fungi are being investigated as a new mosquito control tool because insecticide resistance is preventing successful mosquito control in many countries, and new methods are required that can target insecticide-resistant malaria vectors. Although laboratory studies have previously examined the effects of entomopathogenic fungi against adult mosquitoes, most application methods used cannot be readily deployed in the field. Because the fungi are biological organisms it is important to test potential field application methods that will not adversely affect them. The two objectives of this study were to investigate any differences in fungal susceptibility between an insecticide-resistant and insecticide-susceptible strain of *Anopheles gambiae sensu stricto*, and to test a potential field application method with respect to the viability and virulence of two fungal species

**Methods:**

Pieces of white polyester netting were dipped in *Metarhizium anisopliae *ICIPE-30 or *Beauveria bassiana *IMI391510 mineral oil suspensions. These were kept at 27 ± 1°C, 80 ± 10% RH and the viability of the fungal conidia was recorded at different time points. Tube bioassays were used to infect insecticide-resistant (VKPER) and insecticide-susceptible (SKK) strains of *An. gambiae s.s*., and survival analysis was used to determine effects of mosquito strain, fungus species or time since fungal treatment of the net.

**Results:**

The resistant VKPER strain was significantly more susceptible to fungal infection than the insecticide-susceptible SKK strain. Furthermore, *B. bassiana *was significantly more virulent than *M. anisopliae *for both mosquito strains, although this may be linked to the different viabilities of these fungal species. The viability of both fungal species decreased significantly one day after application onto polyester netting when compared to the viability of conidia remaining in suspension.

**Conclusions:**

The insecticide-resistant mosquito strain was susceptible to both species of fungus indicating that entomopathogenic fungi can be used in resistance management and integrated vector management programmes to target insecticide-resistant mosquitoes. Although fungal viability significantly decreased when applied to the netting, the effectiveness of the fungal treatment at killing mosquitoes did not significantly deteriorate. Field trials over a longer trial period need to be carried out to verify whether polyester netting is a good candidate for operational use, and to see if wild insecticide-resistant mosquitoes are as susceptible to fungal infection as the VKPER strain.

## Background

It is estimated that in 2008 there were 243 million cases of malaria and 863,000 deaths [1]. Clearly, mosquito-borne diseases are still a major health risk, particularly in developing countries. Current mosquito control strategies depend heavily on insecticides but mosquito populations in various disease-endemic countries are developing resistance [2]. Because pyrethroids are the only insecticide class that has WHOPES approval for use on insecticide-treated nets, pyrethroid resistance can seriously hamper vector control activities. Not only does insecticide resistance reduce the capacity to repel and kill mosquitoes, there is also evidence that insecticides can select for certain behaviourally resistant traits, such as earlier mosquito feeding times and earlier exiting from houses with treated nets [3,4]. Furthermore, resistance to some insecticides can confer cross-resistance to other insecticides, notably the organochlorine DDT [5-8]. There is, therefore, an urgent need for alternative tools or strategies that can effectively control insecticide-resistant mosquito populations.

At present biocontrol and biopesticide agents are only operational against mosquito larvae and pupae [9-12]. However, it is the longevity of the adult mosquito that has the greatest impact on the vectorial capacity, and hence transmission intensity, of a mosquito population [13]. Biocontrol agents that target the adult mosquitoes, and to which resistance cannot readily develop, would be useful tools for mosquito control.

The hyphomycetous entomopathogenic fungi *Metarhizium anisopliae *and *Beauveria bassiana *have been used to target pest insects for over a century [14], and have recently been evaluated for mosquito control purposes (see Table 1). These fungi infect mosquitoes through direct contact with the cuticle. The fungal conidia penetrate the mosquito cuticle and grow into the haemocoel where they produce a blend of organic compounds, causing internal mechanical damage, nutrient depletion and death [15]. Lethal effects start to occur three to four days after infection [16-19]. These entomopathogenic fungi are effective at killing both insecticide resistant and insecticide susceptible mosquito populations [20,21]. Furthermore, *M. anisopliae *and *B. bassiana *kill mosquitoes in a slower manner than insecticides kill insecticide-susceptible mosquito populations [17,18,22,23]. To prevent the evolution of resistance it is important to let organisms reproduce before they are killed to allow more than just the individuals with resistance/tolerance genes to contribute to the next generation. It is therefore thought that resistance to fungi will not evolve readily and that they have the possibility to be "evolution-proof" [24,25]. This late acting approach is possible in malaria control where the extrinsic incubation period (EIP) of the parasite is usually three to four gonotrophic cycle lengths (depending on temperature and female susceptibility to infection with *Plasmodium*). Ideally the fungi would kill the mosquito after reproduction had occurred but before she can transmit the malaria parasite.

**Table 1:** Different formulation/substrate application methods used to infect adult malaria vector mosquitoes in previous studies.

Fungus	Formulation	Substrate	Mosquito species	Lab or field	**Ref**.
*B. bassiana*	Dry conidia		*An. albimanus*	Laboratory	[42]

*B. bassiana*	Dry conidia	Agar plate	*An. gambiae s.s.*	Laboratory	[17]

*B. bassiana*	Dry conidia	Plastic tube	*An. gambiae s.s.; An. funestus; An. arabiensis*	Laboratory	[20,21]

*B. bassiana*	Dry conidia	Tissue paper	*An. gambiae s.l.*	Laboratory	[19]

*B. bassiana*	Ondina oil	Cardboard	*An. gambiae s.s.*	Laboratory	[31]

*B. bassiana*	Ondina oil	Paper and netting	*An. gambiae s.s.*	Laboratory	[43]

*B. bassiana*	Ondina oil/ShellSol T	Cage mesh	*An. stephensi*	Laboratory	[36,37]

*B. bassiana*	Ondina oil/ShellSol T	Cardboard pot	*An. stephensi*	Laboratory	[36]

*B. bassiana*	Ondina oil/ShellSol T	Direct application	*An. stephensi*	Laboratory	[36]

*B. bassiana*	ShellSol T	Cardboard	*An. gambiae s.s.*	Laboratory	[31]

*B. bassiana*	ShellSol T	Proofing paper	*An. gambiae s.s.*	Laboratory	[31]

*M. anisopliae*	Coconut oil	Filter paper	*An. stephensi*	Laboratory	[22]

*M. anisopliae*	Dry conidia		*An. stephensi*	Laboratory	[22]

*M. anisopliae*	Dry conidia	Agar plate	*An. gambiae s.s.*	Laboratory	[17]

*M. anisopliae*	Dry conidia	Plastic tube	*An. gambiae s.s.*	Laboratory	[16]

*M. anisopliae*	Enerpar oil	Proofing paper	*An. gambiae s.s.; An. arabiensis*	Laboratory	[23]

*M. anisopliae*	Enerpar/Ondina oil	Black cotton cloth	*An. arabiensis*	Field	[40]

*M. anisopliae*	Ondina oil	Paper and netting	*An. gambiae s.s.*	Laboratory	[43]

*M. anisopliae*	Ondina oil	Cardboard	*An. gambiae s.s.*	Laboratory	[31]

*M. anisopliae*	Ondina oil	Clay pot	*An. gambiae s.s.; An. funestus*	Laboratory	[18]

*M. anisopliae*	Ondina oil/ShellSol T	Cage mesh	*An. stephensi*	Laboratory	[37]

*M. anisopliae*	Ondina oil/ShellSol T	Cardboard pot	*An. stephensi*	Laboratory	[36]

*M. anisopliae*	ShellSol T	Cardboard	*An. gambiae s.s.*	Laboratory	[31]

*M. anisopliae*	ShellSol T	Proofing paper	*An. gambiae s.s.*	Laboratory	[31]

*M. anisopliae*	Sunflower oil	Cotton netting	*An. gambiae s.s.*	Laboratory	[38]

*M. anisopliae*	Sunflower oil	Filter paper	*An. gambiae s.s.*	Laboratory	[16,44]

*M. anisopliae*	Vegetable oil	Black cotton sheets	*An. gambiae s.l.*	Field	[39]

*M. anisopliae*	Vegetable oil	Mud wall	*An. gambiae s.s.*	Field	[38]

Only studies using *B. bassiana *and/or *M. anisopliae *were included in this table

Previous studies have used many different combinations of formulation/substrate (Table 1) to demonstrate the effectiveness of entomopathogenic fungi to infect and kill mosquitoes. However, many of the application methods previously used cannot be deployed easily in the field, either for small-scale tests or for operational vector control. Because fungal spores are biological entities that are affected by the application (formulation/substrate) methods used, it is important to test potential methods that can be used in the field. Many traditional rural African houses are built with open eaves to help air flow within the house. Trials in The Gambia and São Tomé have shown that eaves are important house entry points for *Anopheles gambiae s.l. *[26,27]. Rural African houses also tend to have open windows through which mosquitoes can enter. Eave curtains and insecticide-treated curtains have proven effective at decreasing the numbers of indoor-resting mosquitoes [28] and reducing child mortality [29]. Curtains have a smaller surface area than bed nets, do not come into close contact with humans and would be hung where mosquitoes enter houses. Application of fungal spores onto curtains may, therefore, be a potential application method for mosquito control in the field.

There were two objective of this study, the first was to compare the fungal-susceptibility of an insecticide-resistant and insecticide-susceptible strain of *Anopheles gambiae s.s.*. The second objective was to test a potential application method that could be used in the field. Therefore, *M. anisopliae *and *B. bassiana *conidia were suspended in mineral oil and these suspensions were separately applied onto white polyester netting. The potential of these treated nets to infect and kill *An. gambiae s.s. *SKK (an insecticide-susceptible strain) and *An. gambiae s.s. *VKPER (an insecticide-resistant strain) mosquitoes at different time points after the nets had been treated with fungal conidia was tested using tube bioassays. In addition, fungal viability after application onto the polyester nets was measured.

## Methods

### Mosquitoes

The two mosquito strains used in the bioassays were *An. gambiae s.s. *VKPER and *An. gambiae s.s. *SKK. The SKK strain is an insecticide-susceptible strain originating from Suakoko, Liberia and maintained as a laboratory colony at Wageningen University, The Netherlands, since 1989. The VKPER strain is a pyrethroid-resistant strain that was initially collected from the Kou Valley, Burkina Faso and then selected repeatedly to fix the pyrethroid knockdown resistance (*kdr*) gene. This gene causes target site insensitivity [5] and was first reported in West African mosquitoes in the early 1990s [30]. The VKPER strain has been maintained as a colony at the Centre de Recherché Entomologique de Cotonou (CREC) in Benin, West Africa, for several years. Eggs from this colony were shipped to Wageningen University, The Netherlands, and a colony was started.

Both mosquito strains were subject to standard rearing procedures using tap water in plastic trays (10 × 25 × 8 cm) and fed with Tetramin^® ^fish food daily. Pupae were selected daily and adults were held in standard 30 × 30 × 30 cm gauze-covered cages and fed on a 6% glucose solution *ad libitum*. The larval trays and adult cages were kept in climate chambers held at 27 ± 1°C, 80 ± 10% RH and a 12 hr L:D photoperiod.

### Fungi

Two species of fungi were studied. *Metarhizium anisopliae *var. *anisopliae*, Sorokin isolate ICIPE-30, was produced at Wageningen University, The Netherlands, using solid-state fermentation in aerated packed bed systems with glucose impregnated hemp as a growth substrate. *Beauveria bassiana *Vuillemin IMI 391510 was produced in the laboratory of Penn State University, USA, by initially growing the fungus in a liquid medium and then inoculating autoclaved barley flakes in mushroom spawn bags.

Fungal conidia were dried at ambient temperature (<5% RH) and stored in the refrigerator until use. Dry conidia of *M. anisopliae *and *B. bassiana *were separately suspended in the synthetic isoparaffinic hydrocarbon solvent ShellSol T ™ (Shell, The Netherlands). ShellSol T was selected because the delivery system of fungal conidia suspended in this solvent has been shown to be significantly more virulent to *An. gambiae s.s. *mosquitoes when compared to conidia suspended in other oils [31]. A Bürker-Türk haemocyte counter and light microscope (at ×400) were used to determine accurate conidial concentrations per ml ShellSol T. Fresh suspensions were made for each experimental replicate.

### Net treatment

The netting used was made of white 100% multifilament 150 denier warp-knitted polyester fibres with a mesh size of 12 holes/cm^2 ^(Vestergaard Frandsen, Switzerland). Pieces 15 × 25 cm were used and dipped in the conidia/ShellSol T suspensions resulting in treatment densities of 7.2 × 10^12 ^conidia per m^2^. Control netting was treated with ShellSol T only.

Fungus-treated pieces of netting were held in a climate chamber at Wageningen University under constant conditions of 27 ± 1°C, 65 ± 10% RH, to simulate average climatic conditions of field settings. The viability of fungal conidia (see below) was scored at 1 and 7 days post-treatment, and mosquito bioassays (see below) were run 2 and 7 days post-treatment.

### Tube bioassays

**Figure 1:**
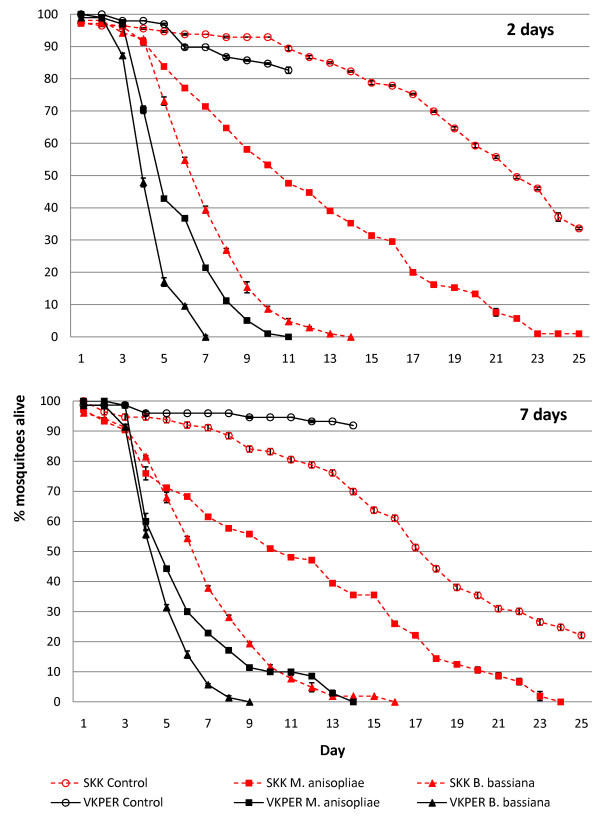
**Effect of entomopathogenic fungal infection on *Anopheles gambiae *survival**. Mean cumulative proportional survival (±SEM) of *Anopheles gambiae s.s. *SKK (dashed red) and *An. gambiae s.s. *VKPER (solid black) mosquitoes after exposure to *Metarhizium anisopliae-*treated (filled squares), *Beauveria bassiana*-treated (filled triangles) or control (open circles) netting 2 (top) or 7 (bottom) days after net treatment.

Separate pieces of control, *M. anisopliae *or *B. bassiana*-treated netting were placed into a tube bioassay set up (8 cm diameter × 15 cm high; see Figure 1E in Farenhorst and Knols [31]) such that the netting covered the inside of the tube. These were stored in a climate chamber at Wageningen University at 27 ± 1°C, 65 ± 10% RH until testing. Tests were carried out in the climate chamber on day 2 and 7 after net treatment.

For the bioassays, the tubes were sealed at both ends with cling film, a surface that mosquitoes do not like resting on. Twenty-five 3-5 day old non-blood fed female *An. gambiae *VKPER or SKK strain mosquitoes were introduced into each tube and exposed to the nets for 1 hr. Four replicates were performed per time point. After the exposure time the mosquitoes were placed into cups and had access to 6% glucose solution *ad libitum*. Every 24 hrs mosquitoes were recorded as being alive if they were still able to fly [32]. Mortality was scored until all the fungus-exposed mosquitoes had died.

Dead mosquitoes were removed daily and checked for fungal infection. Cadavers were dipped in 70% ethanol, for external sterilization, and placed onto moist filter paper in Petri dishes that were then sealed with Parafilm and placed into a 27°C incubator in the dark. After three days it was possible to visually score the proportion of mosquitoes showing fungal infection based on the presence of sporulating fungal hyphae (*M. anisopliae *conidia are green, *B. bassiana *conidia are white).

### Fungal viability

As a measure for conidial viability, the germination of the conidia on a rich agar medium was counted. Either a drop of the conidial suspension or a 1 cm^2 ^piece of the treated netting was placed onto Sabouraud Dextrose Agar (SDA) plates. The SDA plates had 0.001% benomyl added so that accurate germination could be recorded; benomyl is a fungicidal compound that restricts the hyphal growth without affecting germination [33]. These plates were then incubated at 27°C in the dark and germination was scored 24 hrs later using a light microscope at ×400. A conidium was scored as germinated if the germ tube was at least twice the length of the conidium. A minimum of 300 conidia were counted per plate; four replicates of each fungus species/time point were carried out.

### Statistical analysis

For the mosquito survival analysis, differences between the control and fungus-exposed mosquito survival rates were investigated using Cox Regression analysis in SPSS 17.0 [34]. Significant mosquito strain and fungus species effects were further investigated using Cox Regression. Mortality rates were given as Hazard Ratios (HR), which give the average daily risk of dying. Chi-square tests were carried out to investigate the difference between the fungal viability in suspension and on treated nets using SAS 9.1 [35].

## Results

### Tube bioassays

Both *M. anisopliae *and *B. bassiana *were pathogenic to both strains of *An. gambiae s.s.*, with significantly increased mortality in all fungus-exposed/mosquito strain combinations (Table 2). Survival curves for all fungus infected mosquitoes were significantly different from the respective controls both for the mosquitoes exposed two days post net treatment and those exposed seven days after net treatment (Figure 1). Furthermore, *B. bassiana *was significantly more pathogenic than *M. anisopliae *both for SKK (day 2 HR = 3.47, p < 0.0001; day 7 HR = 2.84, p < 0.0001) and VKPER (day 2 HR = 1.89, p < 0.0001; day 7 HR = 1.45, p < 0.05).

**Table 2:** Survival analysis of two strains of *Anopheles gambiae s.s. *exposed to two species of entomopathogenic fungi.

		Days after fungal treatment			
**Fungus**	**Mosquito strain**	**2**		**7**	

*M. anisopliae*	SKK	3.18 (2.31, 4.37)	<0.0001	2.60 (1.94, 3.48)	<0.0001

	VKPER	17.10 (9.68, 30.20)	<0.0001	29.94 (12.72, 70.46)	<0.0001

*B. bassiana*	SKK	11.01 (7.43, 16.32)	<0.0001	7.38 (5.21, 10.45)	<0.0001

	VKPER	32.25 (17.63, 59.02)	<0.0001	43.52 (18.02, 105. 11)	<0.0001

Data show Cox Regression Hazard Ratio outcomes (95% CI)

Statistical p-values are relative to the relevant control

SKK = the insecticide-susceptible *An. gambiae s.s. *SKK strain

VKPER = the insecticide-resistant *An. gambiae s.s. *VKPER strain

There was no significant difference between the control VKPER and control SKK mortalities (HR = 1.63, p = 0.053). However, the insecticide-resistant mosquito strain VKPER was significantly more susceptible to fungal infection when compared to the SKK strain after being exposed to both the two (*M. anisopliae *HR = 4.46, p < 0.0001; *B. bassiana *HR = 3.59, p < 0.0001) and seven day old net treatments (*M. anisopliae *HR = 2.54, p < 0.0001; *B. bassiana *HR = 2.33, p < 0.0001). The number of days since the fungal treatments were applied to the nets caused no significant differences in the mortality of either the SKK (HR = 1.02, p = 0.85) or VKPER (HR = 0.83, p = 0.09) mosquitoes. This indicates that despite the significant drop in fungal viability, the efficacy of the fungal spores in terms of mosquito pathogenicity was equally high seven days after net application.

For both VKPER and SKK mosquitoes, >80% of the dead mosquitoes that were exposed to the fungus-treated netting showed evidence of fungal infection in the form of sporulation. Sporulation rates by themselves do not equate to fungal infection because sporulation varies with many things including fungal dose and virulence of fungal isolate, age of the mosquito and presence of microbial competitors. Although not a perfect indicator for fungal infection, the sporulation of the *M. anisopliae *exposed mosquitoes could be of interest because the viability of the *M. anisopliae *used was so low. For the VKPER mosquitoes that were exposed to the 2 day old *M. anisopliae *treated netting (which had a viability of 13% the day before the bioassay), 82% (80/98) of the mosquitoes showed fungal sporulation. For the mosquitoes exposed to the seven day old *M. anisopliae *net (where the viability was 2%), 84% (59/70) of the mosquitoes showed infection. This was not significantly different from the numbers infected on day 2 (χ^2 ^= 0.02, df = 1, p = 0.65) despite the significant decrease in the viability of the spores on the netting.

### Fungal viability

The viabilities, expressed as the germination rate of fungal conidia, of *B. bassiana *and *M. anisopliae *in the ShellSol T suspensions were 77% and 36% respectively. When the treated polyester net was kept in a climate chamber held at 27 ± 1°C, 65 ± 10% RH for one day, the viabilities of *B. bassiana *and *M. anisopliae *were 71% and 13% respectively. These viabilities had both dropped significantly (*B. bassiana *χ^2 ^= 5.21, d.f. = 1, p < 0.03; *M. anisopliae *χ^2 ^= 192.9, d.f. = 1, p < 0.0001) when compared to the viabilities in suspension. The viabilities of the two fungal species after seven days in a climate chamber were 62% and 2% respectively, for *B. bassiana *and *M. anisopliae. *On top of the significant drop in viability one day after fungal spore application, seven days after net treatment there were significant losses in viability when compared to the day 1 viabilities for both fungal species (*B. bassiana *χ^2 ^= 50.9, d.f. = 1, p < 0.0001; *M. anisopliae *χ^2 ^= 215.5, d.f. = 1, p < 0.0001).

## Discussion

For both species of fungus tested, the insecticide-resistant *An. gambiae s.s. *VKPER strain was significantly more susceptible to fungal infection than the insecticide-susceptible *An. gambiae s.s. *SKK strain. The risk of dying was around 2-4 times higher for VKPER depending on fungal species and age of treatment on the net. A previous study used colony and wild F1 *An. arabiensis *mosquitoes that were exposed to dry conidia of *B. bassiana*. They found no significant differences between the fungal susceptibility of the insecticide-resistant or insecticide-susceptible strains [21]. Another study using dry conidia looked at various *Anopheles *species with various types of insecticide resistance and also found no differences in fungal susceptibility between the insecticide-susceptible and insecticide-resistant strains [20]. The main difference between this and previous studies is that in the present study, mosquitoes were exposed to ShellSol T formulated conidia for 1 hr, whereas the two studies mentioned above exposed mosquitoes to dry conidia for 24 hrs [20,21]. Dry conidia have been shown to kill mosquitoes faster than oil formulated conidia [16]. It is therefore likely that the studies using the 24 hr exposure to dry conidia, whilst good for proving any fundamental principles requiring high fungal infection, caused the mosquitoes to receive such high doses of fungal infection that any subtle strain effects could not be detected.

*Beauveria bassiana *was significantly more virulent than *M. anisopliae *for both mosquito strains. However, it is likely that the difference in virulence is linked to the differing viabilities of the *B. bassiana *and *M. anisopliae *on the treated nets used in this study as this would lead to lower doses being received by the *M. anisopliae*-exposed mosquitoes when compared to the mosquitoes exposed to *B. bassiana. *It is possible that batches of *M. anisopliae *with a higher viability would have similar results to *B. bassiana *because most other studies involving adult mosquitoes that have used these two fungal species have found no differences in their virulence. Blanford *et al *[36] tested a range of oil-formulated fungal isolates of *B. bassiana *and *M. anisopliae *against *An. stephensi *mosquitoes. One *M. anisopliae *isolate used did not prove virulent to mosquitoes, whilst the other had the same virulence as the *B. bassiana *isolates [36]. Similarly, a study examining different application methods found similar virulence levels for oil-formulated *M. anisopliae *and *B. bassiana *when applied to both proofing paper and cardboard, and when different doses of each fungus were applied to proofing paper [31]. When dry conidia were used, Scholte *et al *[17] found that *M. anisopliae *was significantly more virulent to mosquitoes than *B. bassiana *after a three day exposure, although it is unclear what the respective viabilities of the conidia were. Another study using dry conidia found that the virulence of *M. anisopliae *and *B. bassiana *were similar for a range of mosquito species and strains [20].

Broadly speaking, previous fungal studies in the laboratory have used application methods that fall into three categories; dry conidia, using paper as a substrate and using substrates that can directly be used in the field. Of the latter type, studies have been carried out using mosquito cage mesh [36,37], clay pots [18] and cotton netting [38]. In addition to these laboratory studies, field studies in Tanzania have used black cotton cloths [39,40] and direct application onto a mud wall [38]. Of these studies, the fungal viabilities after application onto the substrates were measured for the cotton netting in the laboratory [38] and the black cotton cloths used in the field [39]. In the laboratory, the cotton netting was kept in aluminium foil in the same climate chambers as used in this study, and the viabilities of *M. anisopliae *were 100% in suspension, 94% one day after net treatment and 82% one week after net treatment [38]. For the black cotton sheets used in Tanzania, the *M. anisopliae *viability decreased from 96% in suspension to 95% one day after sheet impregnation and 83% after a week [39]. Unfortunately, due to the different conidial viabilities, doses, exposure times and formulations used for this study and the cotton netting laboratory trial [38] it is not possible to directly compare the relative effect of each type of netting/fungus application method at killing mosquitoes in the laboratory.

When looking at the viability data it appears that the polyester netting/ShellSol T application method would not be a very suitable method for the delivery of viable entomopathogenic fungal spores for mosquito control. However, the virulence data examining the direct effect on mosquito mortality tells a different story. Regardless of time since the treatment of nets with fungi, both fungal species caused significantly increased mortality to both mosquito strains used. The viability of the *M. anisopliae *treated nets was just 2% seven days after net treatment. However, the effectiveness of the fungal treatment at killing mosquitoes did not significantly deteriorate during the length of the trial and high infectivity rates were observed. The differences between the viability and virulence results may be due to the differing abilities of the fungal conidia to germinate on mosquito cuticles and benomyl enriched agar. Whilst benomyl has been shown to not adversely affect the germination of *M. anisopliae *spores when compared to their germination in liquid medium [33], it would be no surprise that such a difference occurs because benomyl is a fungicide and insects are the natural hosts for these fungi.

It is thought that the slow kill speed of entomopathogenic fungi could lead to them being evolution-proof against resistance [25]. This is because any resistance-related genes would be diluted by the genes of susceptible individuals passed onto the next generation before they have succumbed to the fungal infection [25]. For this to be an ethically acceptable strategy for malaria control, the fungi should kill the mosquitoes before the parasite has completed its EIP inside the mosquito. The EIP of malaria parasites can be calculated using the equation [N (days) = 111/(T - t_min_)] from Detinova [41] where T is the mean temperature and t_min _is taken as 16°C [41]. In this study, the experiments were carried out at 27°C; at this temperature the EIP would be 10 days. If entomopathogenic fungi are used on window curtains or bednets, thus targeting host seeking mosquitoes, then a valid assumption would be that a mosquito acquires both fungal and malaria infections at the same time. Given an EIP of 10 days at the experimental temperature, these results show that for the VKPER strain mosquitoes, all mosquitoes would have been killed by *B. bassiana *by this time, and >90% by *M. anisopliae*. In other words, very few fungus-infected VKPER mosquitoes would have survived long enough to transmit malaria. For the less susceptible SKK strain, *B. bassiana *would have killed 90% and *M. anisopliae *just 50% of the mosquitoes by the time the mosquitoes became infectious with malaria. This slower speed of kill found with *M. anisopliae *infected SKK could allow more malaria transmission to occur, but it will also allow more mosquito reproduction, and thus less chance of resistance to fungal infection developing.

## Conclusions

This study shows for the first time that insecticide-resistant *An. gambiae s.s. *VKPER are significantly more susceptible to both fungal species when compared to the insecticide-susceptible *An. gambiae s.s. *SKK. This indicates that entomopathogenic fungi could be used in resistance management and integrated vector management programmes to target insecticide-resistant mosquitoes. Field trials over a longer trial period need to be carried out to see if wild insecticide-resistant mosquitoes are as susceptible as the colony strain used in this trial.

This is the first published study to treat polyester netting with fungal spores. Although fungal viability significantly decreased when applied to polyester netting, the effectiveness of the fungal treatment at killing mosquitoes did not significantly deteriorate during the length of the trial. Following this laboratory trial, studies should be carried out to determine whether polyester netting would be an effective application method for entomopathogenic fungi in the field.

## Competing interests

The authors declare that they have no competing interests.

## Authors' contributions

AFVH designed and undertook the study, analysed part of the data and drafted the manuscript. CJMK analysed part of the data. MF aided in study design and data collection. BGJK supervised the study and aided in study design. WT supervised the study and aided the drafting of the manuscript. All authors read and approved the final manuscript.
